# Limy Bile Syndrome Complicated with Primary Hyperparathyroidism

**DOI:** 10.1155/2015/928217

**Published:** 2015-03-03

**Authors:** Yavuz Savas Koca, Tugba Koca, Ibrahim Barut

**Affiliations:** ^1^Department of General Surgery, School of Medicine, Suleyman Demirel University, Cunur, 32200 Isparta, Turkey; ^2^Department of Pediatric Gastroenterology, Hepatology and Nutrition, School of Medicine, Suleyman Demirel University, Cunur, 32200 Isparta, Turkey; ^3^HPB Surgery Unit, Department of General Surgery, School of Medicine, Suleyman Demirel University, Cunur, 32200 Isparta, Turkey

## Abstract

Limy bile is a relatively rare condition, in which a radiopaque material is visible in the gallbladder on plain radiography or computerized tomography. Cases of complicated hyperparathyroidism are extremely rare. We report a patient with right upper quadrant and epigastric pain and extremity weakness in whom abdominal tomography showed limy bile in the gallbladder and laboratory values showed high levels of serum calcium and parathormone.

## 1. Introduction

Limy bile (LB) is a rare disorder in which the gallbladder and rarely the common bile duct are filled with a paste-like radiopaque material. Volkmann first described the presence of white, calcareous substance in the gallbladder in 1911. Knutsson introduced the term “limy bile” or “milky bile” for this clinical entity [1]. The etiology of LB is unclear, although it may be a long-term complication of total parenteral nutrition. In rare cases of hereditary spherocytosis, primary biliary cirrhosis, or primary hyperparathyroidism, lesions occur simultaneously outside the bile ducts [2].

We report a case of limy bile with cholelithiasis and parathyroid adenoma.

## 2. Case Report

A 40-year-old female patient was admitted with right upper quadrant and epigastric pain and extremity weakness of 15-day duration. She had mild tenderness in the epigastrium and right upper quadrant. No rebound was detected. Physical examination revealed motor power grade 5/5 in the upper and lower limbs. Deep tendon reflexes were normal. Otherwise, there were no other neurological deficits. The only remarkable finding of the physical examination was a nodule in the right lobe of the thyroid.

Laboratory findings were as follows: aspartate transaminase: 11 U/L (8–40); alanine transaminase: 2 U/L (5–35); alkaline phosphatase: 99 U/L (104–338); serum calcium (Ca): 11 mg/dL (8.0–10.0); phosphor: 5.7 mg/dL (2.5–4.5); albumin: 4.3 g/dL (3.5–5.2); parathormone (PTH): 1906 pg/mL (15–65); free T_3_: 2.49 pg/mL (2.5–3.9); free T_4_: 1.1 ng/dL (0.61–1.12); thyroid stimulating hormone: 0.57 uIU/mL (0.34–4.2).

**Figure 1:**
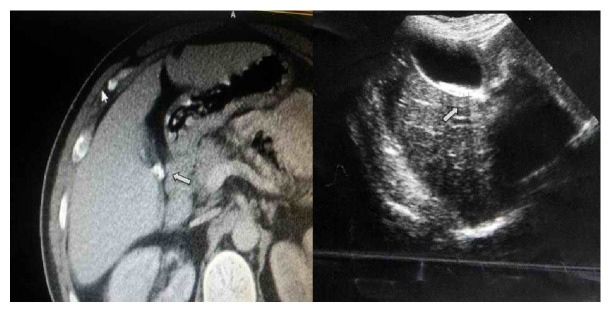
Parathyroid scintigraphy in patient with primary hyperparathyroidism: 99mTc sestamibi SPECT (white arrows).

**Figure 2:**
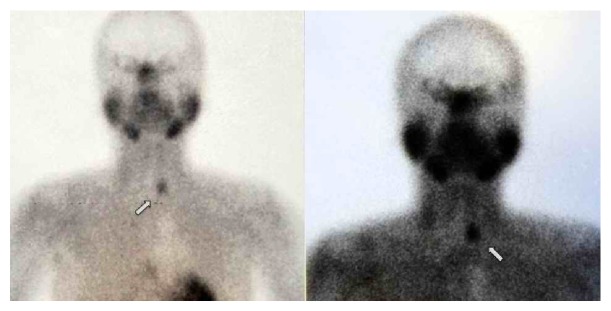
Abdominal CT shows radiopaque material filling the gallbladder and ultrasound shows an acoustic shadow consistent with gallstones (white arrows).

Ultrasound examination of the thyroid showed 27 × 35 × 75 mm left thyroid lobe and 22 × 31 × 63 mm right thyroid lobe. A nodule completely filling the left thyroid lobe containing both heterogeneous necrotic and calcified areas was observed on the ultrasound. Parathyroid scintigraphy showed the left thyroid compatible with parathyroid adenoma (Figure 1). Multiple millimetric stones in the gallbladder were shown on the abdominal ultrasound. Plain abdominal radiography on admission showed a visible gallbladder with calcium density. Abdominal CT showed millimetric radiopaque stones in the gallbladder (Figure 2) and ultrasound showed an acoustic shadow consistent with gallstones. Since the patient had never received radiopaque agents, a diagnosis of LB was made. Cholecystectomy and parathyroidectomy were performed on the patient. The postoperative course was uneventful. Histological examination of the gallbladder showed chronic cholecystitis. The postoperative course was uneventful, and the patient was discharged on postoperative day 4. During the follow-up, PTH at 48 pg/mL, serum phosphor at 4.7 mg/dL, and calcium at 8.7 mg/dL returned to normal levels. The weakness gradually improved. The diagnosis was confirmed by pathological evaluation.

## 3. Discussion

Prevalence of LB has been reported ranging between 0.1 and 1.7% of patients operated on for biliary lithiasis. Onghena et al. reported a prevalence of 0.27% among 1800 Belgian cholecystectomies [1]. It is more common in females (male : female ratio is 1 : 3), and all ages are affected (from the age of 3 years) but it is more commonly observed after the age of 40 years [2–5]. The patient reported here was a 40-year-old female.

The formation of LB in the gallbladder is not well understood, although it has been suggested that some form of obstruction in the cystic duct or gall bladder neck and the resultant bile stasis are necessary [6, 7]. Under these circumstances, precipitation of calcium carbonate, which is normally present in bile, is the end result of local changes in the gallbladder, but the exact mechanism and the time required for these biochemical changes have not been fully elucidated [7]. Chronic inflammatory changes of the gall bladder, which are often observed, seem to be nonspecific and probably secondary.

Therefore, the present case was extremely rare to be associated with primary hyperparathyroidism, because serum calcium and PTH levels were high. However, cholelithiasis and inflammation of the gallbladder wall seemed to exist in this case, and it was thought that if serum calcium levels were high for hyperparathyroidism, calcium would be easily deposited and would consist of LB [8]. LB occurred due to primary hyperparathyroidism in the current patient. Histopathological evaluation was reported as chronic cholecystitis and parathyroid adenoma.

The clinical aspect of the syndrome is similar to that of biliary lithiasis. The presence of LB in the gallbladder can be an incidental finding on plain abdominal X-rays of patients with no symptoms at all. Biliary symptoms are, however, present in most patients with LB syndrome. Complications such as acute cholecystitis, pancreatitis, or obstructive jaundice can also be present. Symptoms and complications do not seem to be the result of limy bile but of the associated biliary lithiasis [7, 9]. There were no symptoms of LB detected in the current patient and it was incidentally diagnosed on USG/X-ray.

Plain abdominal CT scans or abdominal X-rays are crucial in the diagnosis. Characteristic findings are the presence of radiopaque material in the gallbladder, the cystic duct and/or the common bile duct, and the exclamation mark sign, which is present when LB and gallstones coexist in the common bile duct. LB may resemble a normally opacified gallbladder following oral cholecystography. However, it must be determined whether the patient has received any cholecystographic contrast medium before a definitive diagnosis of LB can be established [10]. Ultrasonography is much less specific and in most cases simply reveals concomitant biliary lithiasis. Ultrasound showed an acoustic shadow consistent with gallstones in the patients. Since the patient had never received radiopaque agents, the diagnosis of LB was made.

If extensive calcification of the gallbladder wall is present, ultrasonography and abdominal CT are required as they are considered highly specific in differentiating porcelain gallbladder from other calcifying pathologies such as limy bile syndrome. In such cases, complete or partial calcification of the gallbladder wall is easily differentiated from radiopaque material filling the gallbladder [9].

In conclusion, this case is presented as a rare opportunity of encountering LB syndrome complicated with hyperparathyroidism.

## References

[1] Onghena T., de Waele J. J., Vereecken L., van Loon C., La Meir M. (2001). Limy bile and laparoscopic cholecystectomy. *Acta Chirurgica Belgica*.

[2] Itoh H. (2003). Management of limy bile syndrome: no therapy, laparotomy or endoscopic treatment?. *Internal Medicine*.

[3] Lee S. H., Moon J. H., Choi H. J. (2009). Endoscopic management of acute cholecystitis and cholangitis caused by limy bile. *Gut and Liver*.

[4] Sava G., Millot P., Becmeur F., Vaxman F., Grenier J. F. (1988). Limy bile syndrome. Study of a case with double localization in the gallbladder and common bile duct. *Gastroenterologie Clinique et Biologique*.

[5] Cooke M. (1968). Limy bile. *Proceedings of the Royal Society of Medicine*.

[6] Naryshkin S., Trotman B. W., Raffensperger E. C. (1987). Milk of calcium bile. Evidence that gallbladder stasis is a key factor. *Digestive Diseases and Sciences*.

[7] Peroux E., Geffroy Y., Potet J. (2012). Unenhanced computed tomography to identify intrahepatic and extrahepatic limy bile. *Clinical Gastroenterology and Hepatology*.

[8] Takatori Y., Yamauchi K., Negoro Y. (2003). Limy bile syndrome complicated with primary hyperparathyroidism. *Internal Medicine*.

[9] Sasaki T., Kato D., Matsuoka N., Yamashita Y. (2010). Limy bile syndrome complicated by obstructive jaundice. *International Surgery*.

[10] Ballas K. D., Alatsakis M. B., Rafailidis S. F., Psarras K., Sakadamis A. K. (2005). Limy bile syndrome: review of seven cases. *ANZ Journal of Surgery*.

